# Health Professionals’ Experience Using an Azure Voice-Bot to Examine Cognitive Impairment (WAY2AGE)

**DOI:** 10.3390/healthcare10050783

**Published:** 2022-04-22

**Authors:** Carmen Moret-Tatay, Hernán Mario Radawski, Cecilia Guariglia

**Affiliations:** 1 MEB Laboratory, Faculty of Psychology, Universidad Católica de Valencia, 46100 Valencia, Spain; hradawski@hotmail.com; 2 Department of Psychology, Sapienza University of Rome, 00185 Rome, Italy; cecilia.guariglia@uniroma1.it; 3 Cognitive and Motor Rehabilitation and Neuroimaging Unit, IRCCS Fondazione Santa Lucia, 00179 Rome, Italy

**Keywords:** Azure, health professionals, voice-bot design, cognitive impairment

## Abstract

Virtual Assistants (VA) are a new groundbreaking tool for screening cognitive impairment by healthcare professionals. By providing the volume of data needed in healthcare guidance, better treatment monitoring and optimization of costs are expected. One of the first steps in the development of these items is the experience of the healthcare professionals in their use. The general goal of the current project, WAY2AGE, is to examine healthcare professionals’ experience in using an Azure voice-bot for screening cognitive impairment. In this way, back-end services, such as the ChatBot, Speech Service and databases, are provided by the cloud platform Azure (Paas) for a pilot study. Most of the underlying scripts are implemented in Python, Net, JavaScript and open software. A sample of 30 healthcare workers volunteered to participate by answering a list of question in a survey set-up, following the example provided in the previous literature. Based on the current results, WAY2AGE was evaluated very positively in several categories. The main challenge of WAY2AGE is the articulation problems of some older people, which can lead to errors in the transcription of audio to text that will be addressed in the second phase. Following an analysis of the perception of a group of thirty health professionals on its usability, potential limitations and opportunities for future research are discussed.

## 1. Introduction

Digital technology provides unprecedented big data algorithms for a better understanding of human functioning [1], offering opportunities in different fields, including healthcare. After the current pandemic, health professionals may have experienced an increase in demand for work and new measures, particularly in the treatment of chronic diseases, such as cognitive impairment in older adults [2,3].

While providing supportive services for healthcare, in-home monitoring systems have become a valuable tool for elderly people to make them independent as much as possible [4], more precisely, considering that monitoring the progression of dementia is increasingly important for our society [5,6]. Of note, for cognitive interventions to be effective, therapy must be initiated before extensive tissue damage has occurred, such as when the disease reaches the dementia [7]. Thus, early detection provides the opportunity to intervene to slow disease progression as well as to look for other activities to improve the cognitive reserve [3,4]. On the other hand, healthcare professionals deserve innovative tools to lighten their workload. In this context, systematic reviews show higher levels of burnout when healthcare workers are physically, emotionally, and mentally exhausted, even concerning interest [8,9].

Nowadays, the conventional approach for detecting and diagnosing cognitive impairment involves the clinic-based administration of neuropsychological tests, which are usually performed only in response to patient or family concerns [10,11]. For decades, the most popular screening test among healthcare professionals [12] to examine cognitive impairment has been “Minimental” (MMSE) [13]. Table 1 describes its items and required actions, which can be classified into five components (Orientation, Registration, Attention and Calculation, Recall, and Language) [14]. It should be noted that those who have severe sensory deficits will need and adaptation [15,16], since the result may be misleading (due to a so-called “false positive”). A similar situation might happen for illiterates [17], and differences have been described across different educational levels [18]. MMSE has a strong language component that, according to the literature [19], by adding verbal fluency tests, improves the assessment accuracy of patients with Alzheimer’s disease scoring in the nondemented range.

Other screening tools such as MMSE have been developed in healthcare settings. However, different drawbacks, which might be subjects of innovation, have been also described in the field. Some of the most popular ones are described as follows [20]: (i) Its procedure and administration might be demanding for healthcare institutions as it requires trained clinical staff for administration [21]; (ii) These assessments are based on cross-sectional moments [22]; (iii) The cutoff score might fail to take account of prior clinical information [23]; (iv) These tools are generally unable to detect the more subtle decline in functional ability at the early stages. Nevertheless, MMSE has been adapted to many languages [24,25] has been administered in other scenarios, such as telemedicine ones [26]. However, to our knowledge, the literature is limited regarding the use of Virtual Assistants (VAs) for this propose [27].

Once again, considering that language can be used as an early marker of cognitive decline [28,29], VAs are a new groundbreaking reality. In no case do these technologies replace health professionals, but they provide them with systematized data as a complement to current methods. VAs are artificial intelligence-based software agents, popularized in almost a decade by phone integrated items. The number of virtual assistants has grown since then, with other products such as Amazon’s Alexa, Google’s Assistant and Microsoft’s Cortana. Considering older adults as the final agent of interest in this field, one should bear in mind that the barriers in the adoption of digital devices, such as computers, smartphones and tablets, are related to screens, keyboards, or touch screens to enter data or commands [30]. These devices require reasonable levels of vision and manual dexterity, which can be very demanding for older people [31]. In contrast, voice-powered smart speakers, and, particularly, virtual assistants can avoid these limitations, as they rely on users’ speech and hearing functions.

One example might be Amazon Echo, publicly launched in 2016, being the first ever voice-controlled smart speaker powered by Alexa. The use of this gadget can help in the daily life of a visually impaired elderly person [8,9]. With an Internet connection, its users can listen to news, music, radio channels and audio books. They can also check the time, set timers and alarms, organize personal calendars, search for data and shop online, all with voice commands alone. With other smart home devices installed, they can control other linked systems, all without the need for physical contact. For the first time, a visually impaired person with no previous experience with computers can use these high-tech devices simply by speaking to them.

Providing the volume of data needed in healthcare guidance will result in better treatment monitoring and optimization of costs [10,11]. They will also make VA more accessible, as well as user-friendly, for the older adult population [12]. To bridge this gap between need and opportunity, WAY2AGE is proposed as an interdisciplinary and cutting-edge approach for innovation in early cognitive impairment assessment by health professionals. This project is a pilot phase regarding health professionals’ experience in using an Azure voice-bot to examine cognitive impairment in older adults. Based on the current results, a second phase on more specific aspects of the evaluation of older people will be carried out, involving a different target population and language analysis. However, prior to this second phase, it is considered essential to analyses the experience of health professionals, without which it would not be possible to move on to further steps. In this way, WAY2AGE aims to examine healthcare professionals’ experience on a voice-bot adaptation of traditional tests for screening cognitive impairment and opening a new field by using Model Driven Engineering (MDE) techniques [13].

## 2. WAY2AGE Proposal

The proposed voice assistant is based on Azure cognitive services. By using this Voice Service, the developers might create natural, human-like conversational interfaces for their applications and experiences, including the healthcare field. More precisely, the Voice Assistant Service provides a fast and reliable interaction between a device and an assistant implementation by combining Direct Line Speech (via Azure Bot Service) to add voice functionality to bots, and Custom Commands for voice control scenarios. In this case, a voice-bot is proposed for the assessment of early cognitive impairment by a healthcare professional, using a website hosted in the Microsoft Azure cloud. Back-end services, such as the ChatBot, Speech Service and databases, are provided by the cloud platform Azure (Paas). 

**Figure 1:**
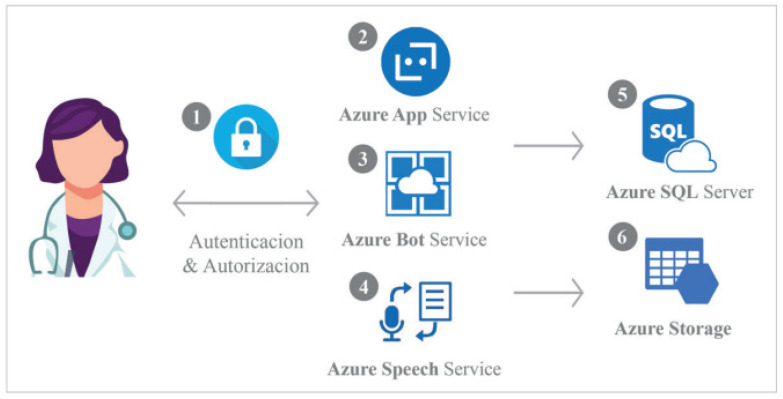
WAY2AGE voice-bot architecture for the piloting (data recruitment) within Azure and its underlying main actions. A digital device is used to activate WAY2AGE by the health professional to implement the cognitive impairment assessment. Image by the company Conectart.

Most of the underlying scripts are implemented in Python, Net, JavaScript, and open software. As the implementation of the NLP algorithm uses several intermediate technologies that are interconnected, such as Microsoft Speech Service, the version control system choose to record changes. The WAY2AGE architecture structure for the pilot phase, measuring professionals’ experience, is depicted in Figure 1.

1. The user (a health care professional) accesses the WAY2AGE application and identifies themselves in the system. The credentials are stored in a database for security reasons. Role-based authorization is controlled by the application.
2. Once healthcare professionals are logged in, they can create new sessions or consult results and recordings.
3. Healthcare professionals access the Bot Service page where the Bot Service interacts with older adults under assessment via text and voice.
4. The Speech Service interprets the older adult’s words and transforms them into text, recording the session in MP3.
5. The text results are recorded in the database by recording the code and date as well as each answer.
6. MP3 files are uploaded to the storage space linked to the database record.

It should be noted that WAY2AGE is designed to be manipulated by healthcare professionals. Once users are logged in, they can create new sessions, repeat, or avoid questions or consult results and recordings. In the current phase, the project is focused on adapting key points on the analysis of cognitive impairment; however, the quality of the data will allow more advanced analyses. 

## 3. WAY2AGE Question Definitions

This section describes how the parameters in Table 1 and other tools were adapted from verbal fluency, as well as the underlying code. Microsoft Bot Framework facilitates the communication between the client’s browser (using Javascript), the channels (Direct and Speech via sockets) and the main core that controls the Dialog. With the help of all these technologies, we can focus on the conversational part. A Waterfall Dialog is used to define a sequence of steps, allowing the bot to guide a user through a linear process (as the flow is linear and in a cascade style, designed to work within the context of a *component dialog)*.

# First, the evaluator assigns an alphanumeric code to maintain the privacy of the end user. This information can be typed or dictated for transcription.

Each time the bot sends a message to the user, it also adds an instruction to leave open the microphone.

[InputHints.ExpectingInput]

…(welcomeText,"es-ES-ElviraNeural","es-ES"),InputHints.ExpectingInput), cancellationToken);

In this code, there is also a voice indicating the language and the type (female). 

# Second, the recording begins. This is step is carried out for two main reasons: (i) in the case of possible articulation problems of the participant that are not correctly transcribed by the algorithm, this allows a subsequent double check; (ii) this is performed for further analysis in future steps based on Natural Language Processing (NLP). For ethical reasons, it is essential that the end user is aware of this step and all their rights.

The recording of the entire session is managed by a Javascript code and RecordRTC.js library, and then attached to the chat by the user. In future versions, these interactions will be automated.

# The first question, “*How do you feel today?*”, is an open-ended question to find out the emotional state of the end user. This will allow to monitor the mood and in future analysis work developed around sentiment analysis. It should be noted that the relationship between geriatric depression and dementia is complex as depressed individuals may indicate a prodromal state of dementia [15]. In this way, the aim was to separate mood disorder from cognitive disorder.

… Prompt = MessageFactory.Text("¿Cómo se siente hoy?", null, InputHints.ExpectingInput)

}, cancellationToken);}

# The second question, “*What did you do yesterday and what are your plans for tomorrow?*”, deals with episodic assessment or time orientation, specifically recent past and prospective memory. This could be considered as an adaptation of the temporal dimension described in widespread tests, such as MMSE [16]. Each time the user starts talking, the system translates as much as possible via speech-to-text and Cognitive Services and sends the response to the bot automatically every 1.5 s, unless the user wants to hear the question again or move on to the next question.

if (!resultado.Result && promptContext.Recognized.Succeeded && !promptContext.Recognized.Value.ToLower().Contains("ripete la domanda")) {

promptContext.Options.Prompt.Speak = "<speak version=\"1.0\"></speak>";

AuxText = AuxText + promptContext.Recognized.Value;

promptContext.Recognized.Value = AuxText;

# The third question, “*What is today’s date and what day of the week are we on*?”, measures temporal orientation and, as in the second question, this could be considered as an adaptation of the temporal orientation dimension described in widespread tests, such as MMSE [16].

# The fourth question, “*Where are we and how old are you?*”, attempts to measure spatial orientation and autobiographical memory adapted from MMSE from its Spanish version MEC [17].

private static async Task<DialogTurnResult> Step5Async(WaterfallStepContext stepContext, CancellationToken cancellationToken) {

stepContext.Values["Step4"] = (string)stepContext.Result;

            return await stepContext.PromptAsync(nameof(TextPrompt),

               new PromptOptions

               {

                   Prompt = MessageFactory.Text(" ¿En qué lugar estamos y cuántos años tiene? ", null, InputHints.ExpectingInput)

               }, cancellationToken);

      }

# The fifth question, “Where are we and how old is it?”, as well as the sixth question, “What is the name of the previous president?”, as in the previous question, is used to measure spatial orientation and autobiographical memory adapted from MMSE from its Spanish version MEC [17].

# The seventh question, “*List for one minute all the names of animals you know*”, attempts to test verbal fluency through an adaptation of the Controlled Oral Word Association (COWA) test from the previous literature [18]. This strategy aims to create a synergy between MMSE dimensions and verbal fluency measures described in the previous literature [19].

In this case, the bot registers the time that the question was asked:

StartTime = DateTime.Now;
stepTimer =true;

and is used to control each interaction until the timeframe is over.

if (stepTimer) {

        var minute = DateTime.Now - StartTime;
        if (minute > TimeSpan.FromMinutes(1) {
          stepTimer = false;
          promptContext.Recognized.Value = AuxText;
          return Task.FromResult(true);

        }

# Question eight is part of an adaptation of the common set-ups for the Brown–Peterson task [19]. In this way, five stimuli are presented. Subsequently, in question nine, an interference task, counting backwards from three-to-three numbers, is performed. Finally, in the last question, we are asked to recall the stimuli of question eight. This allows us to evaluate the working memory in a similar way to MMSE.

## 4. Health Professionals’ Experience

As healthcare workers are the main users of WAY2AGE, this study was approved by the institution’s ethics committee (UCV/2020-2021/163). Regarding the participants’ characteristics, the mean age was 34.90 (SD = 5.05), ranging from 25 to 46 years old. A total of 16.67% were men, while 83.33% were women. Regarding the health profession, 6.67% were Medical Practitioners, 3.33% Nurses, 20% Occupational Therapists, 60% Psychologists and 10% Speech Therapists. The criteria for inclusion of these participants was from them to be in contact with the older adult population in their work and know/use the screening tools for cognitive impairment.

After performing a simulation task on WAY2AGE cognitive screening, a sample of 30 healthcare workers volunteered to participate by answering a list of questions adapted from the previous literature [32], described as follows:

*¿Cómo ha sido la experiencia? (How was your experience?). Answer: 1 (Very bad experience), 2 (Bad experience), 3 (Neutral), 4 (Good experience) and 5 (Very good experience).*

*¿Es WAY2AGE fácil de usar? (Is WAY2AGE easy to use?). Answer: 1 (Very complicated), 2 (Complicated), 3(Neutral), 4 (Easy) and 5 (Very easy).*

*¿Cree que WAY2AGE facilita la evaluación cognitiva? (Do you think WAY2AGE facilitates cognitive assessment?) Answer: 1 (Strongly disagree), 2 (Disagree), 3(Neutral), 4 (Agree) and 5 (Strongly agree).*

*¿Utilizaría esta herramienta en su trabajo? (Would you use this tool in your work?). 1 (Strongly disagree), 2 (Disagree), 3(Neutral), 4 (Agree) and 5 (Strongly agree).*

**Figure 2:**
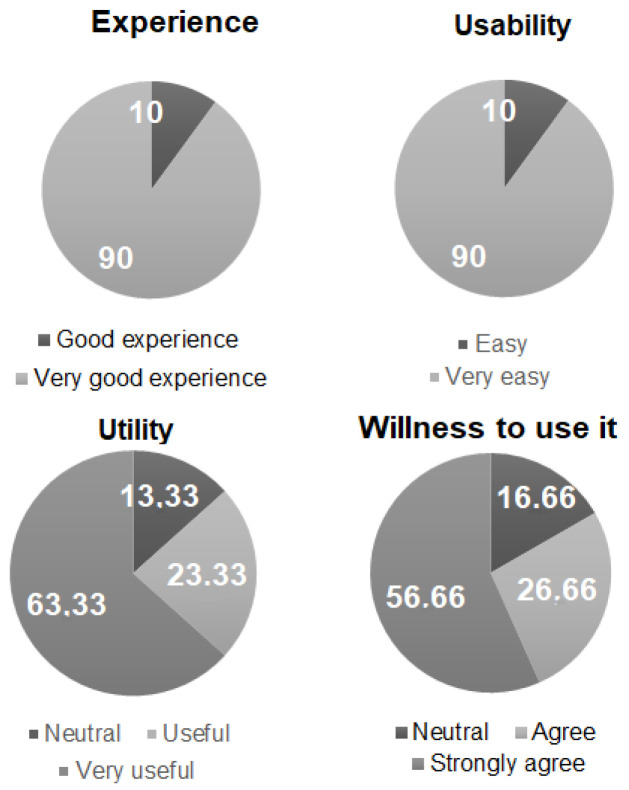
Survey results regarding WAY2AGE usability.

Results are depicted in percentages for Figure 2. WAY2AGE was evaluated very positively in several categories, with a range of 3–5 points. 

Qualitatively, most participants reported that they would like the tool to have automatic data analysis. Moreover, they valued the fact that the session was recorded on MP3 very positively. This allows the healthcare professional and the technicians to check for problems in the transcription from audio to text, as well as any articulation problems of the older adult participant that might result in a barrier for transcription.

## 5. Future Lines of Research: Second Phase

The current phase is a pilot study on the experience of health professionals in a simulated presentation. In the next phase, given the current results, WAY2AGE will be tested on end-users in a second phase, with older adults. WAY2AGE will provide a score of accuracy after each question as well as an analysis of language components. In the first case, WAY2AGE scores will be analyzed as well as the traditional screening tools. To make sure that models can distinguish between diagnostic groups, areas under the ROC curve (AUC) will be used as an indicator. In the second case, language processing components will be analyzed using techniques such as NLP. In this way, results could be filtered through the specific NLP algorithm and analyzed under NLP phases, including lexical (structure) analysis, parsing, semantic analysis, discourse integration, and pragmatic analysis.

The main challenge of WAY2AGE is the articulation problems of some older people, which can lead to errors in the transcription from audio to text. To control this limitation, the results will be recorded in MP3, not just transcribed. This will provide the opportunity for a post-analysis of the recorded discussion and assessment of the impact of this point.

## 6. Conclusions

Health professionals deserve innovative tools and data quality for their assessments. In this work, the architecture for the adaptation of a cognitive assessment test to a voice-bot was shown, which could be of interest in the field. To our knowledge, the literature is scarce on this topic, despite the opportunities for innovation that this field offers [29]. Through the development of instruments such as WAY2AGE, their situation is expected to improve, and ultimately that of older people. In sum, it is expected that senior individuals have an improved quality of life, as well as to improve health decision making in cognitive assessment and inherent AI regulation policies, showing these results as the need of privacy protection [33]. Particularly, an adaptation for the Azure Bot resource that allows to register a bot with the Azure Bot Service was presented. This could be of interest in different fields, such as healthcare. Individuals can build, connect and manage bots to interact with users wherever they are, from the app or website to Teams, Messenger, and many other channels. With regard to ethics, the voice-bot can be designed to manage sensitive data, such as WAY2AGE, restricting who can log in and use the bot [34]. After adapting the main dimensions of cognitive impairment assessment from relevant tests in the field, such as MMSE in its Spanish version or COWA, to implement verbal component assessment, this service was evaluated by a group of health professionals. In relation to the four items of interest, the participants seemed to consider the bot in very positive terms. However, there are underlying questions about its usefulness and the automation of data analysis. The next steps analyzing NLP work in older adults, as final users, might bring promising results. The early detection of impairment though NLP is a methodology soundness that provides the opportunity to slow disease progression as previous results have found interesting markers of impairment though writing [35,36]. Other studies have found possible markers of spontaneous language, but none of them conducted their analysis through VA [28,37,38]. 

## Figures and Tables

**Table 1:** MMSE description adapted from Galasko et al. [19].

Measure	Action Required
Orientation for time	Year, Season, Month, Date and Day
Orientation for place	State, Country, City, Building and Floor
Registration	Repetition of three words
Attention/Calculation	Subtraction of a number from a given digit
Recall	To recall the three words in the repetition phase
Naming	To name two common objects
Repetition	Repetition of a sentence
Three-Stage verbal command	To follow instructions with a piece of paper
Written command	Performing an action by understanding a written sentence
Writing	To write a spontaneous sentence
Construction	To draw interlocking pentagons

## Data Availability

The data presented in this study are available on request from the corresponding author.
